# Construction of High Density Sweet Cherry (*Prunus avium* L.) Linkage Maps Using Microsatellite Markers and SNPs Detected by Genotyping-by-Sequencing (GBS)

**DOI:** 10.1371/journal.pone.0127750

**Published:** 2015-05-26

**Authors:** Verónica Guajardo, Simón Solís, Boris Sagredo, Felipe Gainza, Carlos Muñoz, Ksenija Gasic, Patricio Hinrichsen

**Affiliations:** 1 Centro de Estudios Avanzados en Fruticultura (CEAF), Los Choapinos, Rengo, Chile; 2 Instituto de Investigaciones Agropecuarias, INIA Rayentué, Rengo, Chile; 3 Facultad de Ciencias Agronómicas, Universidad de Chile, Santiago, Chile; 4 Department of Agricultural and Environmental Sciences, Clemson University, Clemson, South Carolina, United States of America; 5 Instituto de Investigaciones Agropecuarias, INIA La Platina, Santiago, Chile; Wuhan Botanical Garden of Chinese Academy of Sciences, CHINA

## Abstract

Linkage maps are valuable tools in genetic and genomic studies. For sweet cherry, linkage maps have been constructed using mainly microsatellite markers (SSRs) and, recently, using single nucleotide polymorphism markers (SNPs) from a cherry 6K SNP array. Genotyping-by-sequencing (GBS), a new methodology based on high-throughput sequencing, holds great promise for identification of high number of SNPs and construction of high density linkage maps. In this study, GBS was used to identify SNPs from an intra-specific sweet cherry cross. A total of 8,476 high quality SNPs were selected for mapping. The physical position for each SNP was determined using the peach genome, Peach v1.0, as reference, and a homogeneous distribution of markers along the eight peach scaffolds was obtained. On average, 65.6% of the SNPs were present in genic regions and 49.8% were located in exonic regions. In addition to the SNPs, a group of SSRs was also used for construction of linkage maps. Parental and consensus high density maps were constructed by genotyping 166 siblings from a ‘Rainier’ x ‘Rivedel’ (Ra x Ri) cross. Using Ra x Ri population, 462, 489 and 985 markers were mapped into eight linkage groups in ‘Rainier’, ‘Rivedel’ and the Ra x Ri map, respectively, with 80% of mapped SNPs located in genic regions. Obtained maps spanned 549.5, 582.6 and 731.3 cM for ‘Rainier’, ‘Rivedel’ and consensus maps, respectively, with an average distance of 1.2 cM between adjacent markers for both ‘Rainier’ and ‘Rivedel’ maps and of 0.7 cM for Ra x Ri map. High synteny and co-linearity was observed between obtained maps and with Peach v1.0. These new high density linkage maps provide valuable information on the sweet cherry genome, and serve as the basis for identification of QTLs and genes relevant for the breeding of the species.

## Introduction

Sweet cherry (*Prunus avium* L., 2n = 2x = 16) belonging to the *Prunus* genus, a member of *Rosaceae* family, is an important tree fruit in Chile. Although Chile is the main producer in the Southern Hemisphere, all sweet cherry cultivars grown in Chile are of foreign origin. Development of sweet cherry cultivars well adapted to local growing conditions that satisfy post-harvest requirements is important for the Chilean industry and represents a significant challenge. Genetic and genomic tools can help mitigate these challenges by improving efficiency and reducing the time necessary to obtain new cultivars. Linkage maps are useful tools in the study of sweet cherry genetics and breeding in that they facilitate the identification and characterization of regions associated with trait(s) of interest and aid development of markers for marker-assisted selection.

For sweet cherry, linkage maps were initially constructed using random amplified polymorphic DNA (RAPD) markers [1] and isoenzymes [2]. Later, maps were constructed mainly using single sequence repeat markers (SSRs) [3–5]. Recently, single nucleotide polymorphisms (SNPs) have been used for linkage maps construction [6, 7]. Cabrera *et al*. [6] used 81 SNPs derived from the Rosaceae Conserved Orthologous Set (RosCOS) [8] for mapping in four sweet cherry F1 populations, representing the first example of high-throughput SNP genotyping assay in sweet cherry. More recently, Klagges *et al*. [7] published high density linkage maps using the RosBREED cherry 6K SNP array v1.0 [9]. This array contains 5,696 SNPs obtained from re-sequencing of 16 sweet and eight sour cherry accessions, with physical position of each SNP based on the peach reference genome (Peach v1.0) as reference. Although the cherry SNP chip represents the variability of a set of cultivars, its fixed number of markers could present a disadvantage in certain crosses. It has been reported that the Peach IPSC 9K Infinium II array [10] did not provide a sufficient number of informative markers for construction of more than several linkage groups in some peach backgrounds [11, 12], showing limitations of the SNP set present on the array, making it suitable for some but not appropriate for other germplasm. However, the lack of linkage groups in the genetics maps obtained from peach crosses is more likely due to identity by descent [13] than array assortment bias. Although SNP markers included in the SNP chips are carefully chosen and present potentially a high number of markers, informativeness of those markers is not ensured for all genetic backgrounds and can hamper the construction of saturated linkage maps.

While previous methods of marker development have been helpful, the recently published “genotyping-by-sequencing” (GBS) method [14], has illustrated a way to identify 1000’s of polymorphic markers for a fraction of the cost of previous methods. GBS allows one to rapidly and completely saturate a linkage map with markers to maximize potential associations with a phenotype. GBS is a simple, highly multiplexed system for constructing libraries for next generation sequencing (NGS). It provides reduced representation sequencing of restriction site associated DNA for rapidly developing markers. The use of methylation-sensitive endonucleases allows targeting lower copy regions with two or three fold higher efficiency [15]. This strategy is especially important because it permits depth coverage of the same regions for all the individuals of a mapping population, and its posterior segregation analysis and linkage maps construction. Saturated linkage maps, constructed using SNPs from GBS, have been published for several plant species such as *Miscanthus sinensis* [16], barley and wheat [17], grapevine [18], blackcurrant [19], rice [20] and alfalfa [21], among others. For the *Rosaceae* family members, genetic maps based on SNPs from GBS have been published for red raspberry (*Rubus idaeus* L.) [22] and apple (*Malus x domestica* Borkh) [23].

In this study, a sweet cherry population derived from the cross ‘Rainier’ x ‘Rivedel’ (n = 166 siblings) was genotyped using SSRs and SNPs via GBS. High density linkage maps for both parents as well as a consensus map were constructed. Filtered sequence reads from GBS were aligned to Peach v1.0 and analysis of distribution along peach scaffolds was performed. These new saturated sweet cherry linkage maps present a valuable set of newly identified SNPs, providing a very powerful tool for quantitative trait loci (QTL) identification and further breeding applications.

## Material and Methods

### Plant material and DNA extraction

The mapping population was developed from 675 one-year-old seedlings obtained from an open-pollinated tree of ‘Rainier’ (female parent) surrounded by several cultivars in a private germplasm collection located at Paine (33°48'13.4"S, 70°40'02.0"W), close to Santiago, Chile [24]. The permission to perform the study on this site was provided by Pablo Canobra. Young leaves of ‘Rainier’, putative pollen donors and all seedlings were collected, immediately frozen in liquid nitrogen and stored at -80°C for later use. Genomic DNA was extracted from the frozen tissue using the DNeasy plant kit (Qiagen, Germantown, MD, USA) according to the manufacturer’s instructions, and quantified using spectrophotometry (Infinite 200 PRO NanoQuant microplate reader; Tecan Tradind AG, Männedorf, Switzerland) and fluorimetry (Quant-iT Picogreen, Invitrogen, Thermo Fisher Scientific, Waltham, MA, USA). The blooming date and location of each tree in the orchard were used as the first filter to propose five probable male parents for the segregating open-pollinated ‘Rainier’ population. These cultivars were ‘Rivedel’ (*S*-alleles haplotype *S1S9*), ‘Vanda’ (*S1S6*), ‘Van’ (*S1S3*), ‘Bing’ (*S3S4*) and ‘Lapins’ (*S1S4*’). The *S*-allele amplification of 675 individual using consensus primers PaConsII [25] was performed and individuals with *S1S9* and *S4S9* haplotypes, representing the most abundant *S*-alleles combination in the population, were selected for further studies. Microsatellite (SSR) markers amplification was performed using seven primer pairs, BPPCT-026 and BPPCT-038 [26]; PMS-30 [27]; PMS-67 [28]; UCD-CH11, UCD-CH12 and UCD-CH21 [29]. Results from both PaConsII and SSR markers identified ‘Rivedel’ as the pollen donor in 232 individuals forming ‘Rainier’ x ‘Rivedel’ (Ra x Ri) population. Initial population was further reduced after subsequent analysis with eight SSRs used for mapping (BPPCT-007 and BPCT-037 [26]; CPPCT-029 and CPPCT-033 [30]; EMPA-004, EMPA-005 and EMPA-013 [31]; UCD-CH14 [29]) and 166 individuals have been selected for GBS analysis and linkage map development. The population is established at Instituto de Investigaciones Agropecuarias, INIA Rayentué, in Rengo (34°19`16.93"S, 70°50`04.15"W), located 110 km South from Santiago, Chile. The mapping population belongs to INIA and do not need any specific authorization to be used for the analysis described in this paper. The field studies did not involve endangered or protected species.

‘Rainier’ is a self-incompatible cultivar (*S1S4*), with early flowering and good productivity. It produces very large fruits, with yellow skin with patches of red blush. Fruit flesh is pale yellow, with excellent firmness, low acidity and sweet flavor. It is susceptible to rain-induced cracking. ‘Rivedel’ is also a self-incompatible cultivar (*S1S9*), with mid to late blooming, early maturity, but with poor fruit set. It also produces large fruit, with dark red skin and flesh, intermediate to firm flesh, with low acidity and medium sweetness. Susceptibility to rain-induced cracking is high to very high.

### SSRs amplification

SSR markers suitable for mapping were selected from a total of 203 SSR markers derived from cDNA and genomic libraries of different *Prunus* species (S1 Table). Parents and two progeny individuals were used to screen markers for polymorphisms and scoring quality. For the majority of the SSR markers, except for EMPA and EMPaS, the polymerase chain reaction (PCR) conditions were as follows: 94°C for 5 min, 35 cycles of 94°C (30 s), 56°C (30 s), 72°C (30 s) and a final extension step of 72°C for 7 min. For EMPA and EMPaS, a touchdown PCR was used as described by Clarke and Tobbutt [31]. PCR reactions were carried out in a total volume of 12 μl, with 20 ng genomic DNA, 0.5 μM of each forward and reverse primers, 0.2 mM dNTPs, 2.5 mM MgCl_2_, 2.4 μl Colorless GoTaq Reaction Buffer (5×), and 0.25 U GoTaq DNA polymerase (Promega, Madison, WI, USA). PCR reactions were carried out on an XP Cycler thermocycler [Bioer Technology, Hi-tech (Binjiang) District Hangzhou, P. R. China]. PCR products were separated using polyacrylamide gel electrophoresis and visualized by silver-staining [32].

### Genotyping-by-sequencing (GBS)

GBS was carried out at Cornell University Biotechnology Resource Center (BRC; Ithaca, NY, USA), following the protocol described for maize by Elshire *et al*. [14]. As a first quality test, 100 ng of DNA from parents and progeny individuals were digested with FastDigest EcoRI (Thermo Fisher Scientific, FL, USA) using manufacturer’s instructions. Each sample was diluted to 100 ng/ul and submitted to BRC for analysis. Briefly, DNAs from the parents and progeny genotypes were digested individually with *Ape*KI restriction enzyme, which recognizes a degenerate five base-pair sequence (GCWGC, where W is either A or T). This enzyme with partial sensitivity to DNA methylation promotes the exclusion of repetitive regions of the genome in this GBS method [14]. GBS sequencing libraries were prepared by ligating the digested DNA to unique nucleotide adapters (barcodes) followed by standard PCR. Sequencing was performed using Illumina HiSeq2000. Parental DNAs were sequenced redundantly three times to increase the number and accuracy of the called SNPs. The filtered sequence reads were aligned to Peach v1.0 (www.rosaceae.org) using the Burrows-Wheelers alignment tool (BWA) [33] version 0.7.7-r441. SNPs were extracted using the GBS pipeline implemented in TASSEL software [34] and genotypes were called using minor allele frequency (MAF) > 0.05.

The raw sequencing data for individual samples has been deposited in NCBI-SRA and is accessible through the BioProject number PRJNA277041.

### SNPs analysis

SNPs were labeled according to the scaffold in the peach genome (s1 to s8), followed by the physical position in base pairs (bp). Location of each SNP within genic (exonic, intronic and UTR) and intergenic regions was determined using Perl script (www.perl.org) with Peach v1.0 as reference. Order of SNPs along scaffolds was plotted using MapChart 2.2 [35].

As the nomenclature of *Prunus* SNPs has not been standardized yet, the physical position of each SNP was used to identify common markers among this study, SNPs from the RosBREED cherry 6K SNP array v1 [7, 9] and SNPs obtained from 3’UTR sequencing from ‘Bing’ and ‘Rainier’ [36].

### Linkage maps construction

Marker scoring, SNPs, SSRs and *S*-alleles, followed the coding scheme for cross-pollinated population type proposed in JoinMap 4.1 (Kyazma B.V, Netherlands) [37]. In case of SNPs, polymorphic heterozygous markers in only one of the parents were scored either <lmxll> or <nnxnp> and heterozygous markers in both parents were scored as <hkxhk>. SNP markers with more than 10% of missing data were removed. Marker segregation distortion was determined by calculating chi-square (χ²) using JoinMap. Maternal, paternal and consensus map construction was performed using the regression mapping algorithm. For parental maps construction, markers were grouped using a minimum independence LOD score of 8.0 and linkage groups were established at an LOD score of 3.0 to 5.0 and maximum recombination frequency of 0.35 or 0.40, depending on the linkage group. Map distance was estimated using Kosambi mapping function.

Maternal and paternal maps were constructed using three criteria: (i) with markers following Mendelian segregation; (ii) using markers following Mendelian segregation and markers showing segregation distortion (p≥0.05); and (iii) all available markers [those following Mendelian segregation and markers showing segregation distortion (p≥0.0001)]. After comparing the paternal maps, only results from first and second round from regression mapping were considered. Finally, results for maternal and paternal linkage groups constructed with markers from (ii) are presented with the exception of ‘Rivedel’ linkage group (LG) 6. Due to the high degree of skewed markers in this group, results from (iii) are presented. A visual inspection of ordered genotype data was performed using *Data* tabsheet function in JoinMap. False double recombination events within small genetic distances were corrected according to the genotype of the neighboring markers using information from *Genotype Probabilities* [37]. For the construction of the consensus (Ra x Ri) map, markers mapped in both parental maps plus a selection of markers segregating in both parents, class <hkxhk> [markers showing segregation distortion (p≥0.05)], were used. As for parental maps construction, consensus LG6 was constructed using all available markers. The ‘One-step method’ [38] was used for consensus map construction considering the same parameters used for parental maps construction, except a minimum independence LOD score of 12.0 was used for grouping markers. SSR markers mapped in the consensus map were compared to published maps [4, 5] using the position of common SSR markers. Maps were plotted using MapChart 2.2 [35].

## Results

### Genotyping-by-sequencing and number of segregating SNPs identified

Within our *P*. *avium* mapping population, GBS produced between 1,384,008 and 4,277,386 reads per individual, with an average of 2,333,869 reads. From a total of 11,854 SNPs obtained in the progeny (MAF > 0.05), markers that represented more than 10% of missing data in the population and markers with incorrect genotypes (for example, parental configuration AAxAB with BB progeny) were eliminated. Finally, a group of 8,476 high quality informative SNPs were selected for mapping (S2 Table). This group contains 1,950 SNPs (23%) with maternal segregation type <lmxll>, 1,880 (22.2%) with paternal segregation type <nnxnp> and 4,646 (54.8%) with segregation type <hkxhk>, heterozygous in both parents.

Segregation distortion was observed in 56.7% (1,106 out of 1,950) of the markers segregating in the female parent <lmxll> and 68% (1,278 out of 1,880) of the markers segregating in the male parent <nnxnp> (goodness-of-fit ratio 1:1, α = 0.05), as well as in 92.5% (4,299 out of 4,646) of the markers heterozygous in both parents <hkxhk> (goodness-of-fit ratio 1:2:1, α = 0,05).

### Comparative genomics

Analysis of 8,476 high quality SNPs showed their even distribution along the main eight peach scaffolds (S1 Fig). A group of 244 SNPs were located in other scaffolds, but they were not included for analysis. The number of identified SNPs ranged from 757 for scaffold 5 to 1,709 for scaffold 1 (S3 Table). A total of 215 Mb of the peach genome were covered with marker density of approximately one SNP per 25 Kb (Table 1). Gaps were observed in all scaffolds (S1 Fig), with the maximum gap size ranging from 419 Kb (scaffold 2) to 851 Kb (scaffold 5) (Table 1).

**Table 1:** Physical position of sweet cherry SNPs identified by genotyping-by-sequencing.

Scaffold	No. SNPs	Physical position of the first SNP (bp)	Physical position of the last SNP (bp)	Scaffold coverage (bp)^a^	Scaffold coverage (%)^a^	Scaffold coverage (bp) / No. SNPs	Maximum gap (bp)	Last SNP before the maximum gap	First SNP after the maximum gap
**1**	1,709	54,451	46,826,337	46,771,886	99.8	27,368	743,699	s1_24087177	s1_24830876
**2**	1,102	166,103	26,687,741	26,521,638	98.9	24,067	419,113	s2_10459166	s2_10878279
**3**	891	60,528	21,781,726	21,721,198	98.6	24,378	610,099	s3_7797530	s3_8407629
**4**	1,071	123,401	30,112,346	29,988,945	98.2	28,001	623,255	s4_24336804	s4_24960059
**5**	757	317,038	18,460,309	18,143,271	98.1	23,967	851,246	s5_6908686	s5_7759932
**6**	1,173	55,118	28,840,333	28,785,215	99.6	24,540	498,403	s6_14057760	s6_14556163
**7**	873	447,405	22,649,317	22,201,912	97.4	25,432	494,944	s7_741027	s7_1235971
**8**	900	138,739	21,704,360	21,565,621	98.8	23,962	567,809	s8_9725707	s8_10293516
**Total**	8,476			215,699,686					
**Average**					98.7	25,214			

^a^ Scaffold coverage based on length of peach scaffolds [39].

**Fig 1:**
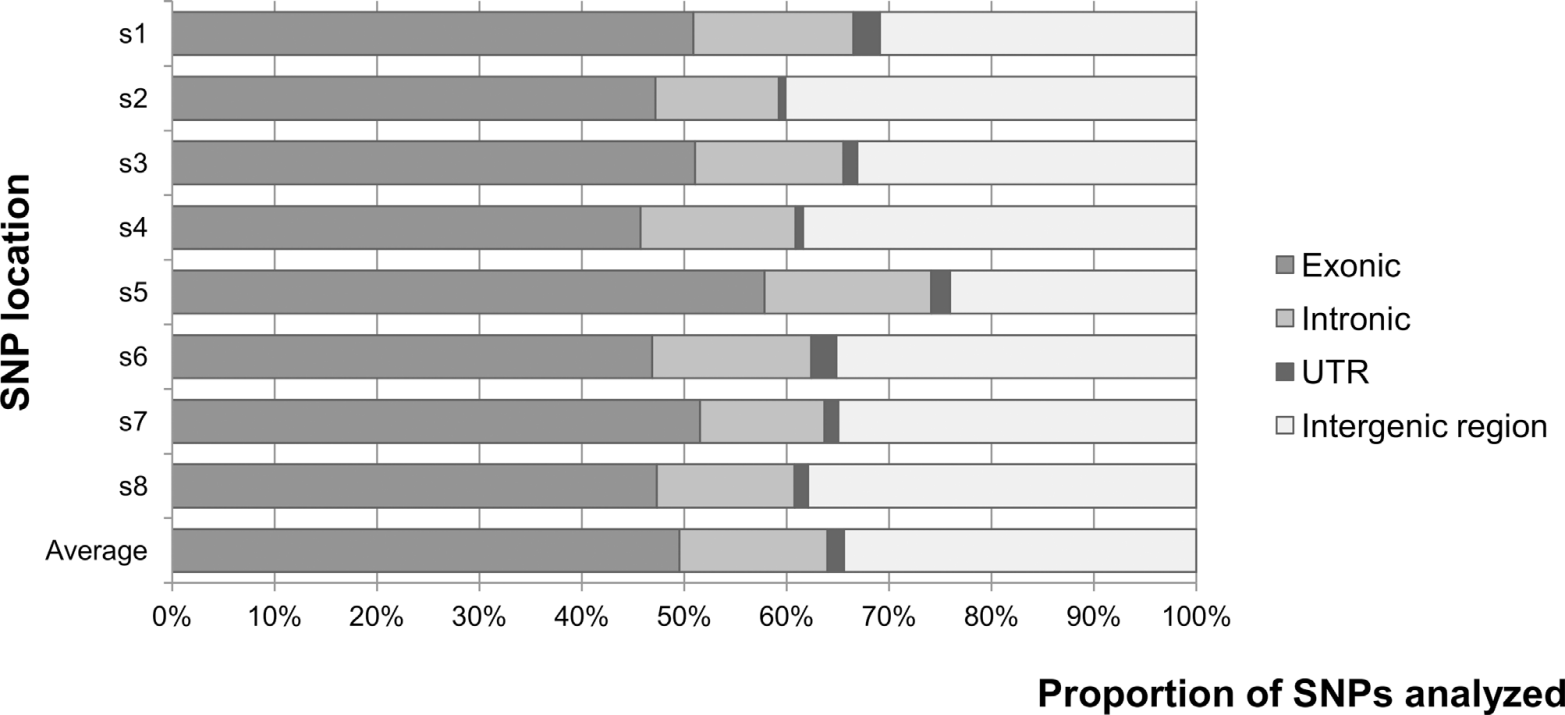
Distribution of SNPs in genic [exonic, intronic, untranslated (UTR)] and intergenic regions using the physical position of each SNP on Peach v1.0 [39]. Figure represents coverage of each SNP category per scaffold and an average of all categories across all scaffolds.

Location of each SNP within genic [exonic, intronic and untranslated regions (UTR)] and intergenic regions, determined by using the physical position of each SNP in respect to the annotated peach genome, is shown in Fig 1 and S4 Table. When all the scaffolds are considered, on average 65.6% of the SNPs were located in genic regions (49.8% in exons, 14.3% in introns and 1.5% in UTR), and 34.4% SNPs were located in intergenic regions. In detail, distribution of SNPs located in exons varied between 45.8–57.9% in scaffolds 4 and 5, respectively; in introns between 12.1% for scaffold 2 and 16.3% for scaffold 5; and in UTR regions between 0.7% for scaffold 2 and 2.6% for scaffold 1 (Fig 1, S4 Table). The highest number of SNPs in genic regions (76.0%) was observed on scaffold 5. Proportion of SNPs located in intergenic region ranged from 24–40.1% in scaffold 5 and 2, respectively.

Physical position of each SNP along peach scaffolds allowed the identification of common markers with previous studies, which also used Peach v1.0 as a reference genome. Out of the 39 SNPs, in common between our study and the cherry 6K SNP array v1 [7, 9], only 16 were mapped in our study. Therefore, due to the low number of common SNPs, comparisons of previously reported linkage maps with those produced in our study were not possible. In addition, no common SNPs were found with the SNPs obtained from 3’UTR sequencing from ‘Bing’ and ‘Rainier’ [36].

### SSRs segregation and mapping positions

In total, 203 *Prunus* SSR markers were evaluated within our Ra x Ri population (S1 Table). Only 169 (83.3%) primer pairs successfully amplified PCR products, and 69 of them were polymorphic. After discarding markers with complex or inconsistent amplicon patterns, a group of 34 SSRs was used for mapping (S5 Table). Out of 34 SSRs, 19 were mapped in ‘Rainier’, 15 in ‘Rivedel’ and 21 in the consensus map (Table 2). No SSRs were mapped in LG3 and LG4 of the ‘Rainier’ map; LG3, LG4 and LG8 of the ‘Rivedel’ map and LG4 of the consensus map. Similar physical position of SSRs common between this study and those utilized by Peace *et al*. [9], confirmed conservation of marker order (data not shown). Furthermore, comparison between the position of SSRs, on linkage groups containing three or more SSRs in the consensus map, in common with previously published PAxPN [*P*. *avium* ‘Napoleon’ (PA) x *Prunus nipponica* accession F1292 (PN)] linkage map [5], revealed similar genetic distances between markers and high degree of colinearity between linkage groups. The only exception was an inversion at the bottom of the LG2 observed in Ra x Ri consensus map (S2 Fig).

**Table 2:** Description of 'Rainier', ‘Rivedel' and consensus linkage maps.

	‘Rainier’					‘Rivedel’					Consensus				
LG	SNPs	SSRs	Length (cM)	Avg. marker distance (cM)	Max. gap (cM)	SNPs	SSRs	Length (cM)	Avg. marker distance (cM)	Max. gap (cM)	SNPs	SSRs	Length (cM)	Avg. marker distance (cM)	Max. gap (cM)
**1**	99	5	139.6	1.3	20.7	104	4	131	1.2	11.1	211	2	138.4	0.6	5.2
**2**	62	4	68.9	1.0	9.3	49	3	62.9	1.2	12.9	120	5	86.5	0.7	5.8
**3**	55	0	73.7	1.3	10.5	36	0	78.4	2.2	29.1	113	1	94.8	0.8	5.8
**4**	13	0	28.8	2.2	10.5	56	0	56.2	1.0	8.5	71	0	70.7	1.0	5.9
**5**	64	5	60.1	0.9	6.3	35	1	60	1.7	19.9	130	5	75.8	0.6	3.8
**6**	78	1	73	0.9	9.6	78	6	80.3	1.0	5.3	137	4	109.6	0.8	8.3
**7**	19	3	38.6	1.8	7.4	79	1	59.4	0.7	6.2	87	3	81.8	0.9	8
**8**	53	1	66.8	1.2	8.0	37	0	54.4	1.5	10.1	95	1	73.7	0.8	4.4
**Total**	443	19	549.5	1.2	10.3	474	15	582.6	1.2	12.9	964	21	731.3	0.7	5.9

LG—linkage group.

### Linkage mapping

**Fig 2:**
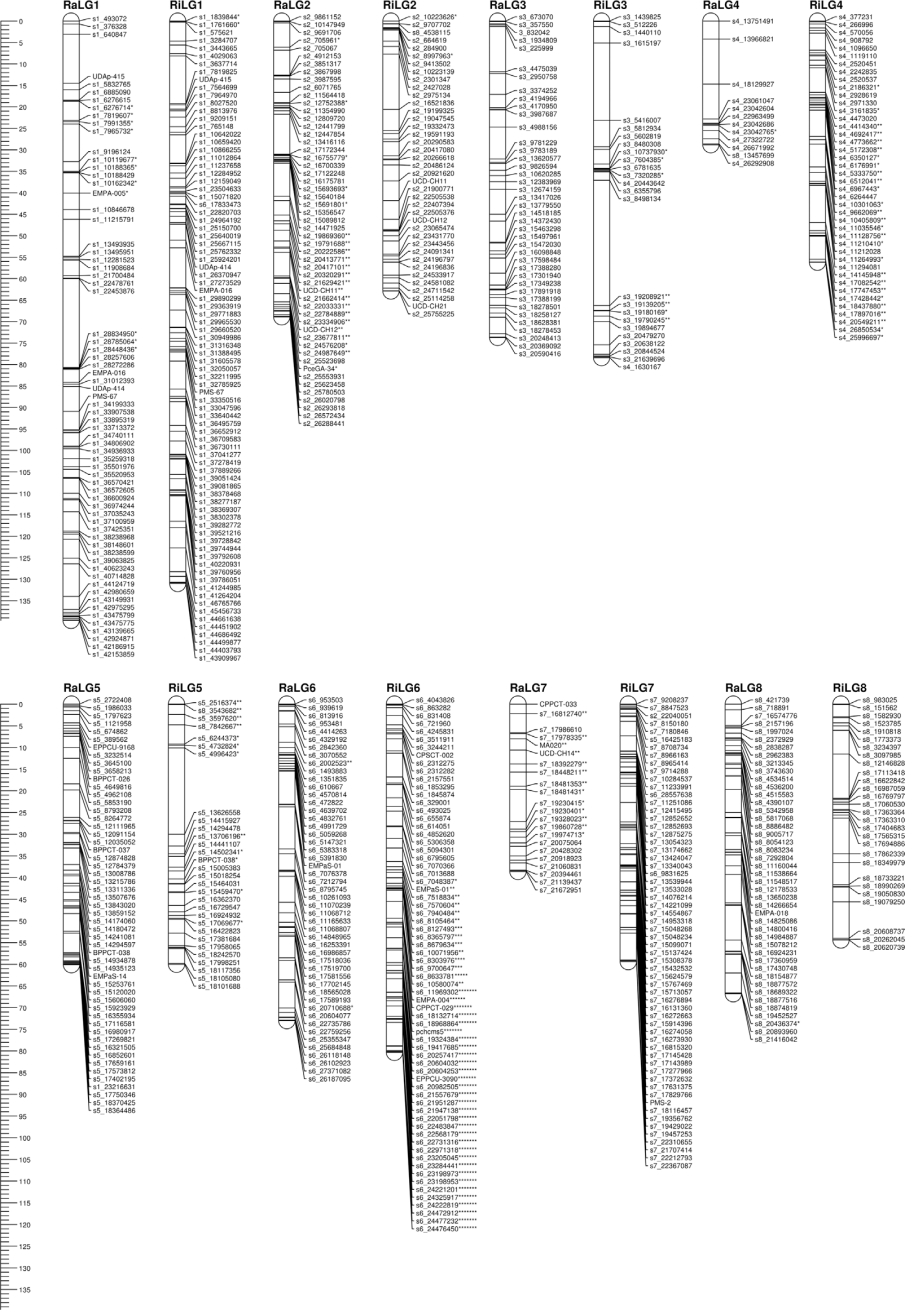
Linkage maps for ‘Rainier’ (Ra) and ‘Rivedel’ (Ri), generated through JoinMap 4.1. Asterisks indicate distortion level of skewed markers where: * = p<0.1; ** = p<0.05; *** = p<0.01; **** = p<0.005; ***** = p<0.001; ****** = p<0.0005; ******* = p<0.0001). Redundant markers have been removed and only unique positions are represented. The scale in centiMorgan (cM) is given at the left side of the figure.

A set of 3,830 SNPs, with 1,950 having maternal segregation type <lmxll> and 1,880 paternal segregation type <nnxnp>, plus 34 SSRs were used for maps construction. Both parental maps were composed of eight linkage groups (Table 2, Fig 2). ‘Rainier’ linkage map comprised 462 markers (443 SNPs and 19 SSRs) covering a total of 549.5 cM. The LG length was variable, with LG1 being the largest, 139.6 cM, and LG4 covering the shortest distance, 28.8 cM. The average marker density was 1.2 cM per marker. Maximum gap size ranged from 6.3 cM in LG5 to 20.7 cM in LG1. ‘Rivedel’ linkage map comprised 489 markers (474 SNPs and 15 SSRs) and covers a total of 582.6 cM, with a minimum and maximum linkage group length of 54.4 cM (LG8) and 131 cM (LG1) and an average marker density of 1.2 cM per marker. Maximum gap size ranged from 5.3 cM for LG6 to 29.2 cM for LG3. The highest number of skewed markers, 58 out of 84 (69%), was mapped in ‘Rivedel’ LG6 (Fig 2).

**Fig 3:**
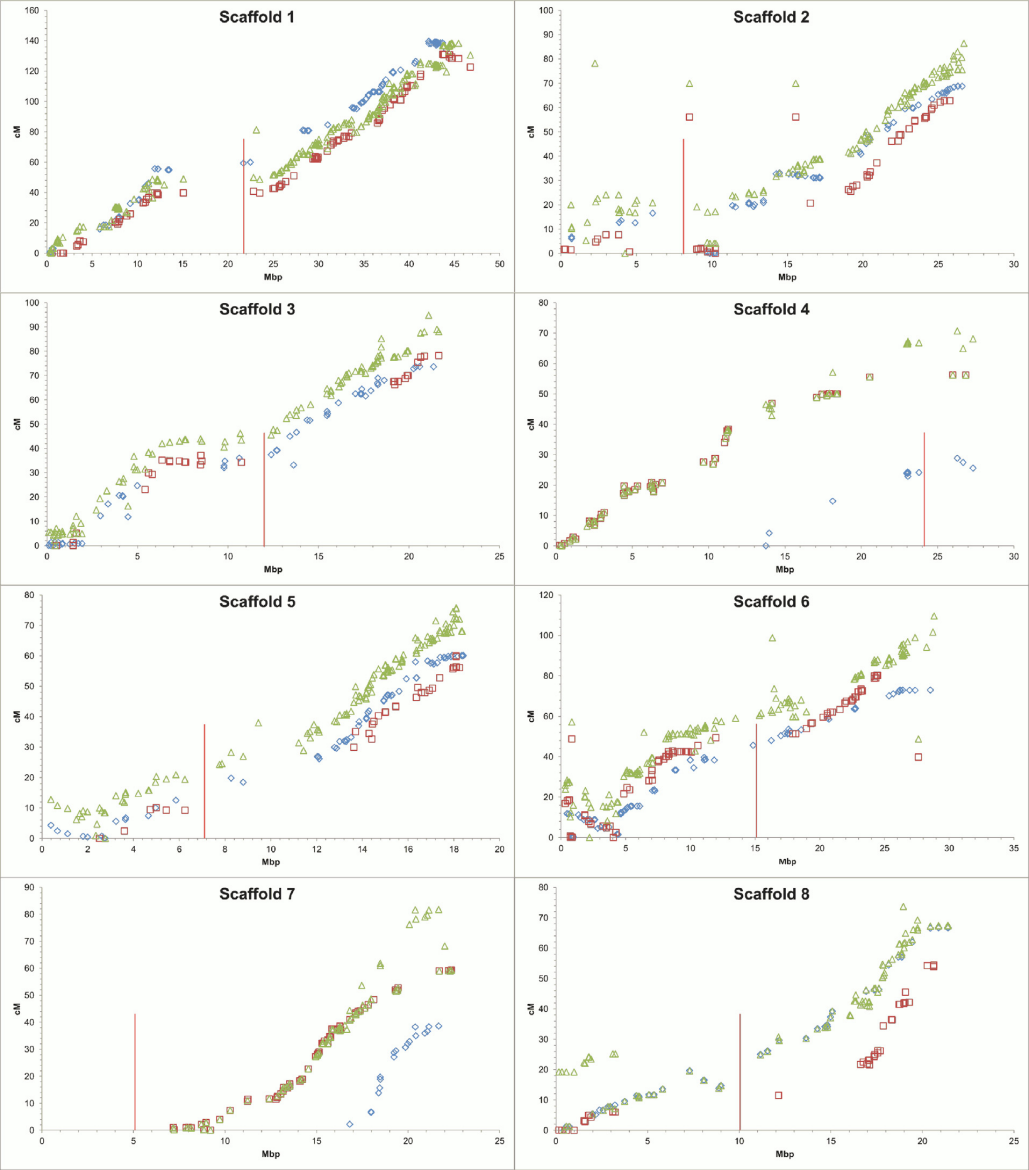
Relationship between genetic and physical distances for each linkage group and scaffold. Physical distance along each scaffold is showed on the horizontal axis (Mbp) and genetic distance is showed on the vertical axis (cM). Location of markers from ‘Rainier’ (blue diamonds), ‘Rivedel’ (green triangles) and consensus (red squares) linkage maps are showed. The vertical bars indicate the putative position of the centromeric regions using information from the Peach v1.0 [39].

Important gaps, observed in certain linkage groups, also represent considerable physical distances between flanking SNPs. For ‘Rainier’, overlapping gaps between genetic and physical maps have been observed in LG2 (9.3 cM with 3.8 Mb), LG4 (9.9 cM with 4.2 Mbp) and LG5 (6.3 cM with 3.9 Mbp), and for ‘Rivedel’, in LG2 (12.9 cM with 12.7 Mbp), LG3 (29.1 cM with 10.7 Mbp), LG4 (8.5 cM with 2.9 Mbp), LG5 (19.9 cM with 8.6 Mbp) and LG8 (10.1 cM with 5 Mbp) (S6 Table). Gaps in linkage groups 1, 3, 5 and 8 (Fig 3) coincide with putative centromeric regions in the peach genome sequence [39].

**Fig 4:**
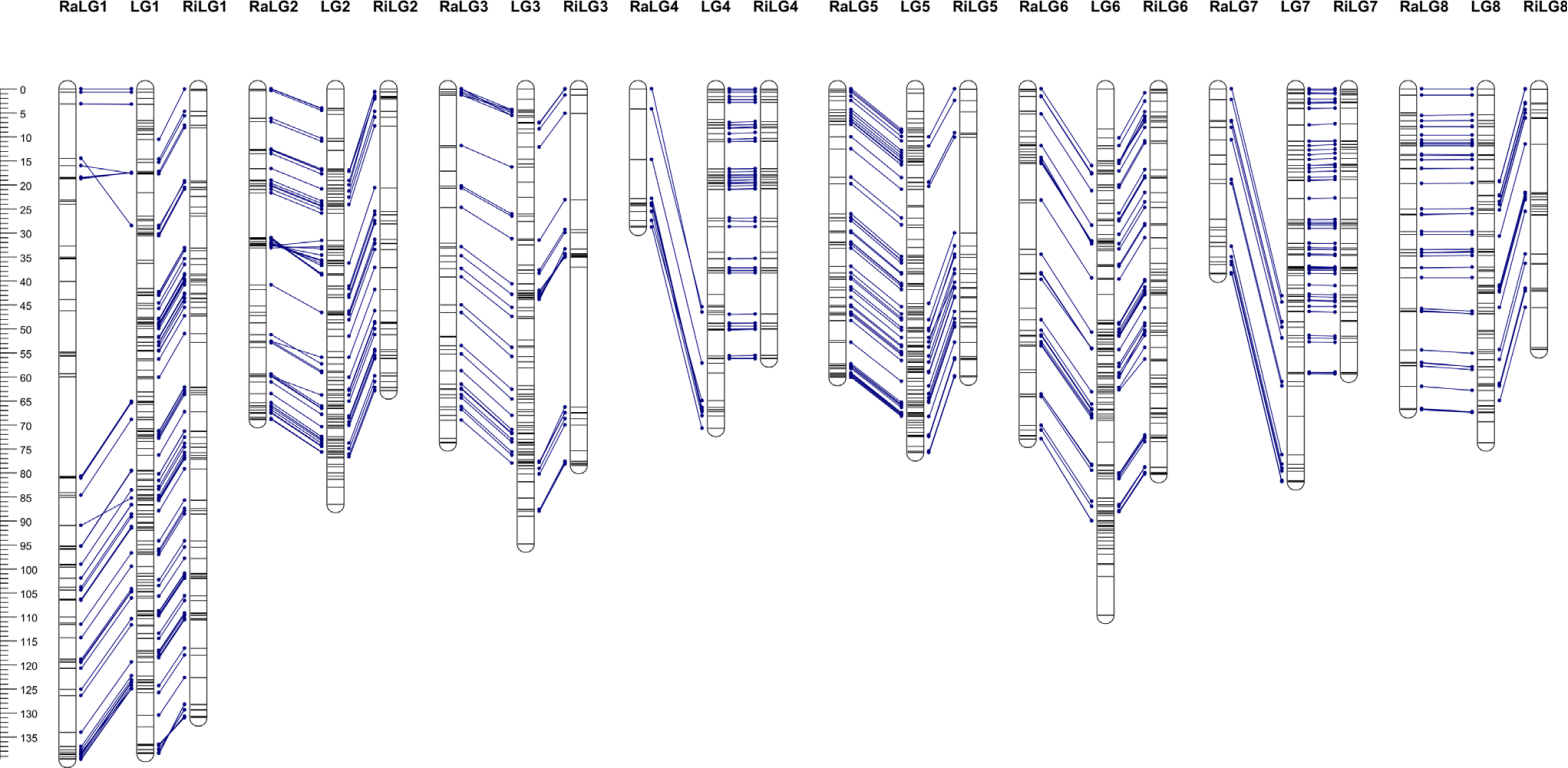
Comparison between parental and consensus linkage maps. Common markers are indicated by blue lines between parental and consensus maps. A scale in centiMorgan (cM) is given at the left side of the figure.

The consensus linkage map, constructed with markers mapped in both parental maps plus a selection of markers segregating in both parents, class <hkxhk>, comprises 985 markers and covers a total of 731.3 cM, with a linkage group length ranging from 70.7 cM (LG4) to 138.4 cM (LG1) and an average marker distance of 0.7 cM per marker (Table 2). Comparison between parental and consensus maps (Fig 4) revealed, in general, good coverage of the consensus map with markers from both maternal and paternal maps. Exceptions are LG1 (34 markers mapped in ‘Rainier’, but not mapped in the consensus map) and LG6 (42 and 33 markers mapped in ‘Rainier’ and ‘Rivedel’, respectively, but not mapped in the consensus map). Only minor changes in markers order were observed for LG1, LG2, LG3 and LG6 (S7 Table).

Mapped SNPs in all three maps, paternal and consensus, were located mainly in genic regions (S8 Table). On average, 81.9%, 78.6% and 80.0% of mapped SNPs were located in genic regions in ‘Rainier’, ‘Rivedel’ and consensus map, respectively. The largest number of SNPs located in genic regions were observed in LG7 (89.5%) of ‘Rainier’, and LG8 (83.8%) of ‘Rivedel’ and consensus map (86.3%).

### Comparison of the physical and genetic maps

**Fig 5:**
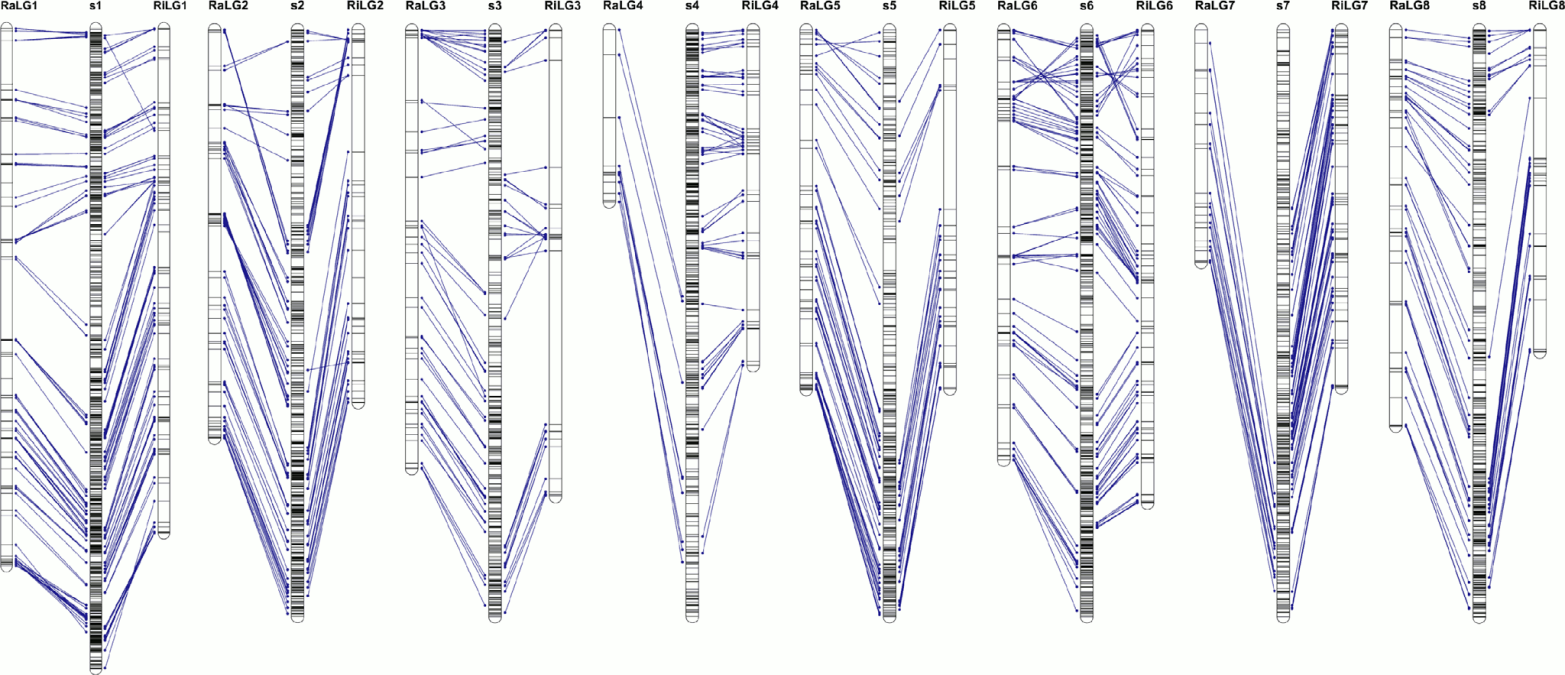
Comparison of the genetic parental maps and physical map constructed using all SNPs identified by GBS for sweet cherry cultivars used in this study (n = 8,476), and ordered according to their physical position in Peach v1.0 [39]. Distances have been proportionally modified to show the results.

In general, linkage positions of all SNPs in the two parental and consensus maps were in agreement with their physical position on the pseudomolecules/scaffolds of Peach v1.0. However, exceptions were observed in both parental maps when compared with the physical map created using all SNPs detected by GBS (Fig 5). For ‘Rainier’, out of 443 mapped SNPs, only 5 (1.1%) were mapped on different linkage groups from their physical position, and for ‘Rivedel’, the same was observed for 14 out of 474 mapped SNPs (3%). Partial coverage of scaffolds was observed for ‘Rainier’ LG4 and LG7, with mapped SNPs only from the bottom region of the corresponding scaffold. The highest homology between the genetic and the physical position was observed for ‘Rivedel’ LG5 and LG7. Inversions of groups of markers in respect to their expected physical position were observed in both parental genetic maps. Five regions of the ‘Rainier’ map, involving 24 markers on LG1, 16 on LG2, eight on LG5, 21 on LG6 and four on LG8, appeared inverted relative to the physical map. Similar results were observed in three regions of the ‘Rivedel’ map, involving 11 markers on LG1, five markers on LG3 and ten markers on LG6 (S9 Table). In addition, the top region of LG2 in both parental maps housed a group of SNPs that according to their physical position should be mapped lower around 9–10 Mbp into the corresponding scaffold.

## Discussion

Genetic studies and molecular breeding approaches require basic genomic resources, such as molecular markers and linkage maps. To develop resources for sweet cherry genetic studies, we used SNPs obtained from genotyping-by-sequencing (GBS) and previously published SSR markers to construct high density linkage maps of sweet cherry. To our knowledge, this is the first report of linkage mapping with SNPs obtained from GBS in cherry and second in *Prunus* species [40]. Parental and consensus maps provide a valuable resource for genetic analysis of sweet cherry, with excellent genome coverage for most of the linkage groups.

Among other GBS advantages, this technology is capable of accessing single-copy gene rich regions as well as regulatory regions, which are important because the latter contain functionally important elements such as promoters [14]. In this study, the availability of Peach v1.0 [39] and genetic collinearity and high synteny among *Prunus* species [3, 4, 6] have been exploited for SNP detection via GBS in sweet cherry. Our results revealed one SNP per 25 Kb nucleotides and, on average, 65.6% of SNPs in genic regions (S4 Table), with most SNPs located in exons. The observed coverage of genic regions is higher than that published for soybean [41], where 39.4% of SNPs were located in genic regions, with 20.7% of them in exons. The high percentage of SNPs located in exons (49.8%) reinforces the usefulness of DNA digestion with an enzyme partially sensitive to methylation, allowing digestion of DNA in less methylated, single-copy gene rich regions of the genome [14]. Considering that approximately 80% of mapped markers are located in genic regions (S8 Table), linkage maps constructed in this study present an important tool for finding candidate genes underlying traits of interest and potential functional markers for use in marker assisted selection.

From a group of 8,476 high quality SNPs obtained by GBS, 1,950 SNPs (23%) were heterozygous in the maternal cultivar ‘Rainier’, and 443 of them (22.7% of 1,950) were mapped in parental map. In the pollen donor ‘Rivedel’, 1,880 SNPs (22.2%) were heterozygous and 474 of them (25.2% of 1,880) were mapped. Although the number of heterozygous SNPs obtained with this technology is higher than reported in Klagges *et al*. [7] for each parent using the RosBREED cherry 6K SNP array v1.0 [9], the percentage of mapped SNPs was lower. From 5,696 SNPs available on the cherry 6K SNP array, the number and percentage of heterozygous SNPs, together with mapped SNPs ranged from 634 (11.1%) in ‘Black Tartarian’, with 384 (60.6%) mapped, to 515 (9.0%) in ‘Lapins’, with 247 (48%) mapped. Differences in percentage of mapped heterozygous SNPs between both studies are possibly due to redundancy of SNPs in our study. We used Regression Mapping [42] algorithm that permits the construction of linkage groups in three rounds by adding loci one by one, starting from the most informative pair of loci [37]. When the three rounds for maps construction were used, the resulting map had higher number of markers compared to the first and second round but marker order was lost because markers were positioned without restrictions. It has been noted previously that the third round map does not produce a good quality final result [37]. Therefore, we used results from first and second round only, giving priority to the quality over the quantity.

Results from GBS confirmed that pollen donor identification was successful because all individuals presented alleles coming from the expected parents. Linkage maps presented in this study were constructed using a mapping population generated from open-pollination of a sweet cherry cultivar ‘Rainier’. Although controlled pollination is the best strategy for obtaining mapping populations with specific trait(s) combination, it takes a long time to develop it. The use of a progeny from an open-pollination of a maternal parent of interest, would bypass the need for development of a progeny from a particular parental combination. It would also speed-up the development of maps, particularly in species with fewer resources available. It would also enable faster QTL discovery, since the open-pollination progeny would be already phenotyped for some of the traits of interest in a given breeding program.

Linkage maps were constructed using markers without distortion and markers showing segregation distortion (p≥0.05) for all linkage groups because higher saturation for linkage groups was not achieved when all markers were used, except for ‘Rivedel’ LG6. A total of 67 (14.5%) and 128 (26.2%) skewed markers were placed on ‘Rainier’ and ‘Rivedel’ linkage maps, respectively. A clustering of loci with skewed segregation ratios was observed on ‘Rainier’ LG2 and ‘Rivedel’ LG4 and LG6, which is in agreement with previously reported clustering of loci with high distortion near the bottom of LG6, in vicinity of the *Prunus* self-incompatibility *S* locus [3, 7].

SSRs developed in different *Prunus* species were mapped together with SNPs. From 203 *Prunus* SSR markers screened, only 34 (16.8%) were used for mapping, which is lower than the 26% reported by Olmstead *et al*. [4]. This is probably due to the low heterozygosity of the parents in this study. A comparison between SSRs mapped in our consensus linkage map and markers from the consensus map published by Clarke *et al*. [5] demonstrated a high degree of collinearity between both maps, with an inversion on the distal part of LG2. This difference observed may be due to markers mapped in the Ra x Ri consensus map that are heterozygous in both parents (<hkxhk>). It was not possible to perform comparisons with other previously published maps because of the low number of shared markers, considering both SSRs and SNPs. Only 16 SNPs in common between GBS SNPs and the RosBREED cherry 6K SNP array v1 were mapped in this study. This could be explained by a low matching between genome regions represented in both studies. Peace *et al*. [9] indicated that only 14.1% of raw cherry reads from most conserved regions between cherry and peach genomes were used for chip construction. Cherry SNP array was constructed from SNPs discovered using whole genome sequencing data from several sweet and sour cherry cultivars while in our study genetic material from only two different cultivars is the base for SNP detection. In addition, the Tassel SNP discovery pipeline [43] obtains only SNPs that are different between the sweet cherry samples included in this study using Peach v1.0 as a reference genome. Given that the peach genome sequence is obtained by sequencing di-haploid ‘Lovell’ [39], and inherent differences between peach and sweet cherry genomes, coupled with the limitations of Infinium II assay, may have contributed to the detection of low common SNPs between our GBS study and 6K cherry SNP [9]. On the other hand, a possible reason for low number of common SNPs between this study and those obtained from 3’UTR sequencing of ‘Bing’ and ‘Rainier’ [36] could be due to the low coverage of these regions using GBS in our population (1.5%, considering 3’ and 5’ UTR regions).

Parental maps constructed in this study (549.5 cM for ‘Rainier’ and 582.6 cM for ‘Rivedel’) are smaller than parental maps reported earlier for this species (711 cM for ‘Emperor Francis’ [4]; 719.4 cM for ‘Black Tartarian’, 788 cM for ‘Kordia’, 619.4 cM for ‘Regina’ and 610.1 cM for ‘Lapins’ [7]) and comparable to the map reported for ‘New York 54’ (565.8 cM) by Olmstead *et al*. [4]. The length of the consensus map (731.3 cM) is similar to previously published sweet cherry consensus maps (680 cM for the interspecific PAxPN consensus map [5]; 799.4 cM for the consensus map constructed with RosCOS [6]; 752.9 cM for BTxK and 639.9 cM for RxL populations [7]). Certain linkage groups exhibited significant differences between parental maps, such as LG4 and LG7 being smaller in ‘Rainier’ than in ‘Rivedel’. In particular, ‘Rainier’ LG4 was almost half of the length of ‘Rivedel’ LG4. Detailed analysis revealed that the physical position of SNPs mapped on LG4 and LG7 only represent the bottom segment of the respective peach scaffolds. Olmstead *et al*. [4] obtained short linkage groups for LG3, LG4 and LG5 of the ‘New York 54’ parental map and suggested it is due to the overall low level of heterozygosity in this cultivar. Consequently, results obtained in this study could be explained by the low level of heterozygosity in the top of both ‘Rainier’ linkage groups.

Several inversions of SNP order from their predicted physical orientation were observed in both parental maps (Fig 5 and S9 Table). Observed inversion in LG1, LG5 and LG6 coincide with previously reported observations in ‘Black Tartarian’, ‘Kordia’, ‘Regina’ and ‘Lapins’ LG1; ‘Black Tartarian’ and ‘Lapins’ LG5 and ‘Kordia’ and ‘Lapins’ LG6 [7]. Klagges *et al*. [7] indicated that these discrepancies correspond with minor assembly errors of the peach genome. Inversions not previously reported were observed in the central region of ‘Rainier’ LG2 and LG8, and ‘Rivedel’ LG3, and correspond with misassembled regions of Peach v1.0 (I. Verde personal communication). The observed discrepancies could also be due to low coverage of these regions in previous maps or errors in the assignment of marker order. In addition, markers mapped in the top region of LG2 in both parents could correspond to genome duplication because their physical position indicates that they are located in the region between 9–10 Mb.

Results of this study, where only 1.1% of ‘Rainier’ mapped markers and 3% of ‘Rivedel’ mapped SNPs were located on different linkage groups from their projected physical map, support previously reported high level of synteny between sweet cherry and peach genomes. Availability of the sweet cherry genome reference sequence [44] in the future will enable alignment of reads obtained in this study to cherry genome. It is expected that genomic variants among both genomes will enable identification of new SNPs by alignment of reads to the regions of the sweet cherry genome. Genomic variants such as SNPs, InDels, duplications, inversions and translocations have been reported in peach by Fresnedo-Ramírez *et al*. [45] by comparing three new peach genome sequences with Peach v1.0, and by Di Genova *et al*. [46] in grapevine by sequencing the ‘Sultanina’ (a table grape cultivar) genome and comparing it with the *Vitis* reference genome PN40024. New alignments analyses using information obtained from GBS in this study and the sweet cherry reference genome sequence will help answer some of the results obtained in this study and give us valuable information about this *Prunus* genome.

In conclusion, we have constructed, to the best of our knowledge, the first cherry high density linkage maps using SNPs obtained from GBS and microsatellite. These high quality SNPs are located mainly in genic regions of the eight scaffolds of the peach reference genome, and most mapped markers belong to this group. Due to the small number of shared SNPs with other studies, only mapped SSRs were compared with previously published maps revealing a comparable order. Nevertheless, the high number of SNPs identified in this study presents a valuable set of new SNPs identified in sweet cherry that would be useful for genetic studies in the future. Order of SNPs along linkage groups confirmed a high synteny level between sweet cherry and peach genomes, with only small discrepancies. New studies on synteny between both species will be possible when the sweet cherry genome becomes available. New linkage maps constructed in this study provide valuable information on the sweet cherry genome, as the basis to identifying QTLs and genes relevant for the breeding of the species.

## Supporting Information

S1 Fig Distribution of 8,476 SNPs detected in ‘Rainier’ x ‘Rivedel’ population across eight peach scaffolds. Black lines represent physical position of each SNP according with Peach v1.0 [39]. “S1[1]” and “S1[2]” correspond to Scaffold 1, which was divided in two parts for a better visualization of the results. Distance between markers is presented in Mbp.
(TIF)

S2 Fig Comparison between four Ra x Ri linkage groups and the corresponding linkage groups of PAxPN [*P*. *avium* ‘Napoleon’ (PA) x *P*. *nipponica* F1292 (PN)] [5] using common SSR markers. Genetic distances are given in centiMorgan (cM).
(TIF)

S1 Table Microsatellite markers used in sweet cherry linkage maps construction. (XLSX)

S2 Table SNPs identified in this study using genotyping-by-sequencing. (XLSX)

S3 Table Number and segregation type of informative SNPs in Ra x Ri progeny by scaffold. (XLSX)

S4 Table Distribution of SNPs in genic (exonic, intronic and UTR) and intergenic regions. (XLSX)

S5 Table Microsatellite markers used for mapping. (XLSX)

S6 Table Description of the overlapping gaps between physical (bp) and genetic (cM) parental maps. (XLSX)

S7 Table Markers mapped in 'Rainier', 'Rivedel' and consensus maps, including the linkage group and map position to which they were located. (XLSX)

S8 Table Summary of mapped and genic SNPs, and proportion of mapped SNPs in genic regions in parental ('Rainier', 'Rivedel') and consensus maps. (XLSX)

S9 Table Discrepancies between physical (according to Peach v1.0) and genetic position of mapped markers. (XLSX)
