# Supplementary figures and images for: Construction of High Density Sweet Cherry (Prunus avium L.) Linkage Maps Using Microsatellite Markers and SNPs Detected by Genotyping-by-Sequencing (GBS)
Source: PLoS One. 2015 May 26;10(5):e0127750. doi: 10.1371/journal.pone.0127750 (PMC4444190; doi:10.1371/journal.pone.0127750)

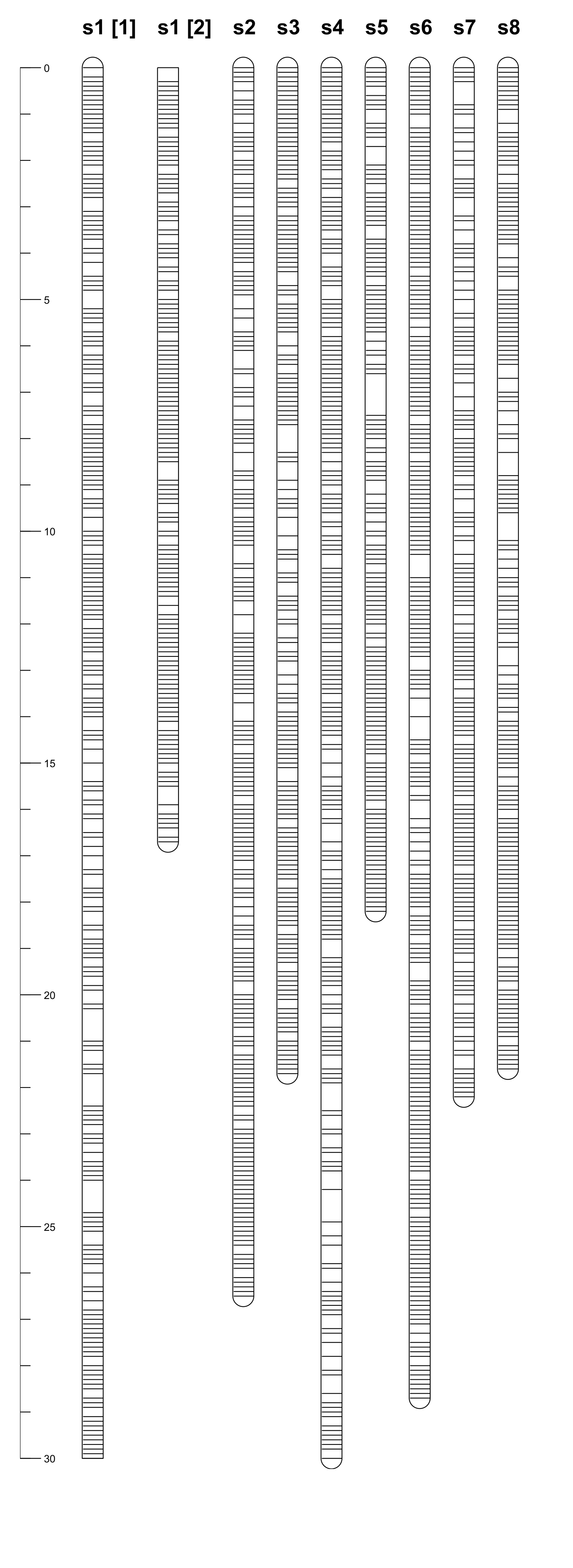

Supplement: S1 Fig — Black lines represent physical position of each SNP according with Peach v1.0 [39]. “S1[1]” and “S1[2]” correspond to Scaffold 1, which was divided in two parts for a better visualization of the results. Distance between markers is presented in Mbp. (TIF) [file pone.0127750.s001.tif]

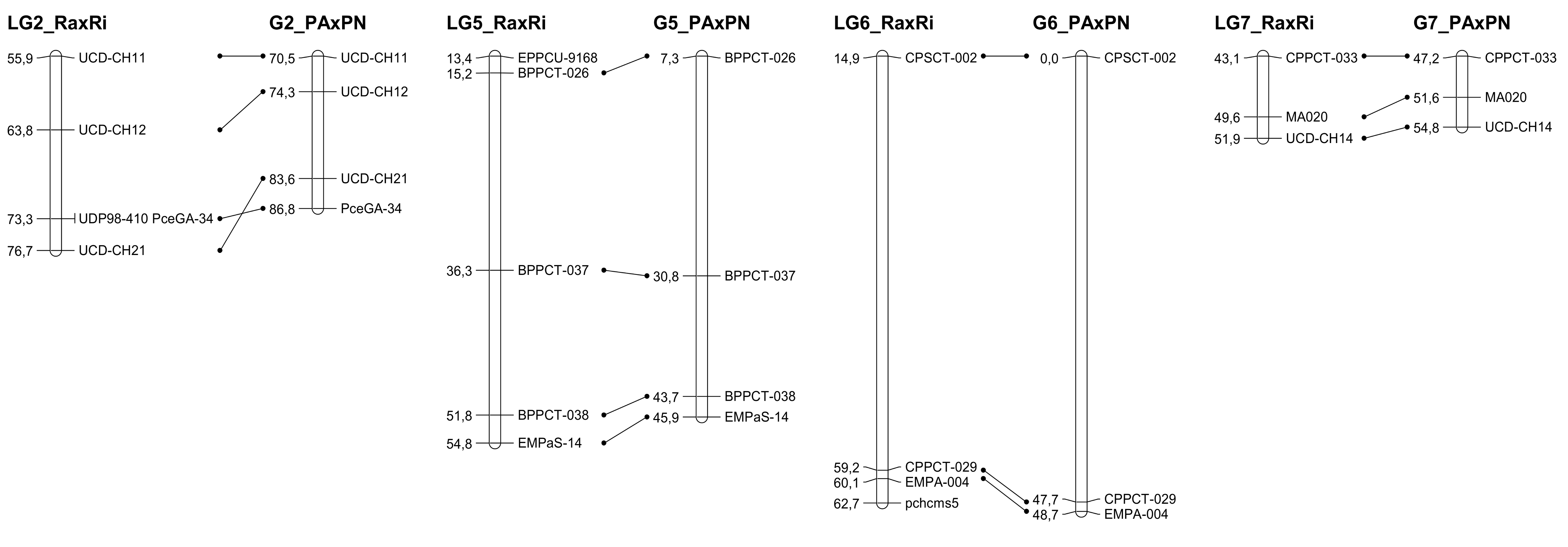

Supplement: S2 Fig — Genetic distances are given in centiMorgan (cM). (TIF) [file pone.0127750.s002.tif]
